# Why Clinicians Don’t Report Adverse Drug Events: Qualitative Study

**DOI:** 10.2196/publichealth.9282

**Published:** 2018-02-27

**Authors:** Corinne M Hohl, Serena S Small, David Peddie, Katherin Badke, Chantelle Bailey, Ellen Balka

**Affiliations:** ^1^ Centre for Clinical Epidemiology and Evaluation Vancouver Coastal Health Research Institute Vancouver, BC Canada; ^2^ Department of Emergency Medicine University of British Columbia Vancouver, BC Canada; ^3^ Vancouver General Hospital Emergency Department Vancouver, BC Canada; ^4^ School of Communication Simon Fraser University Burnaby, BC Canada; ^5^ Department of Pharmaceutical Sciences Vancouver General Hospital Vancouver, BC Canada

**Keywords:** adverse events, pharmacovigilance, drug safety, adverse drug reaction, adverse drug event, electronic health records, information and technology, medication reconciliation, qualitative research

## Abstract

**Background:**

Adverse drug events are unintended and harmful events related to medications. Adverse drug events are important for patient care, quality improvement, drug safety research, and postmarketing surveillance, but they are vastly underreported.

**Objective:**

Our objectives were to identify barriers to adverse drug event documentation and factors contributing to underreporting.

**Methods:**

This qualitative study was conducted in 1 ambulatory center, and the emergency departments and inpatient wards of 3 acute care hospitals in British Columbia between March 2014 and December 2016. We completed workplace observations and focus groups with general practitioners, hospitalists, emergency physicians, and hospital and community pharmacists. We analyzed field notes by coding and iteratively analyzing our data to identify emerging concepts, generate thematic and event summaries, and create workflow diagrams. Clinicians validated emerging concepts by applying them to cases from their clinical practice.

**Results:**

We completed 238 hours of observations during which clinicians investigated 65 suspect adverse drug events. The observed events were often complex and diagnosed over time, requiring the input of multiple providers. Providers documented adverse drug events in charts to support continuity of care but never reported them to external agencies. Providers faced time constraints, and reporting would have required duplication of documentation.

**Conclusions:**

Existing reporting systems are not suited to capture the complex nature of adverse drug events or adapted to workflow and are simply not used by frontline clinicians. Systems that are integrated into electronic medical records, make use of existing data to avoid duplication of documentation, and generate alerts to improve safety may address the shortcomings of existing systems and generate robust adverse drug event data as a by-product of safer care.

## Introduction

Adverse drug events are harmful and unintended consequences of medication use [1]. They include adverse drug reactions and harmful events related to drug dosing, noncompliance, treatment failures, ineffective drugs, drug interactions, the inappropriate use of drugs, and events due to errors [1]. Up to 70% are deemed preventable [2-4], yet they remain a leading cause of emergency department visits and hospitalizations [2,5,6], indicating a need to strengthen postmarket surveillance and drug safety research to develop more effective prevention strategies [7,8].

Patient safety and quality improvement initiatives have focused on reducing medication errors, which can be investigated using root cause analysis. However, most clinically significant adverse drug events are *not* error-related [2,9] and require the identification of patient-, medication-, provider-, and system-level factors that can be used to develop and implement prevention strategies. These activities are limited by lack of robust representative population-level data on adverse drug events [8].

Drug regulators and researchers rely on few data sources and methods to ascertain adverse drug event outcomes including administrative data, disease- or drug-specific registries, and paper-based or electronic records mined using triggers consisting of diagnostic codes, words, phrases, or laboratory values suggesting an adverse drug event occurred. These data sources and methods generate incomplete data lacking important details. For example, a validation study comparing trigger methods to prospectively collected data found that only 2% to 15% of events were identifiable using trigger methods compared to prospectively collected data and these lacked important details [10-12]. Spontaneous reporting systems put in place by drug regulators to stimulate reporting by clinicians suffer from reporting rates of less than 5%, even in jurisdictions where reporting is mandatory [8,13,14]. Underreporting contributes to delays until sufficient data accumulate for drug safety signals to be detected and undermines comparative risk assessments that would be useful when several treatment options exist [7]. Finally, underreporting in spontaneous reporting systems is more likely to affect older, commonly prescribed drugs, shifting the focus away from them even though older drugs cause a high burden of disease and should continue to be the focus of drug safety research [2,5,15,16]. Active surveillance systems in which trained staff follow up with patients to investigate previously identified safety signals are currently used to gather high-quality information on suspect events [14]. Such systems require dedicated staff and funding, have thus far focused on high-risk drugs and specialized patient populations, and may be less practical for widespread surveillance.

The uptake of electronic medical records provides opportunities for adverse drug event reporting to be integrated into point-of-care documentation. Repeat exposures to medications that previously caused harm are common in elderly populations and cause repeat adverse drug events [17,18]. If functional adverse drug event reporting software could be integrated into electronic medical records, adverse drug event reports could be used to generate patient-level alerts to prevent reexposures to medications that previously caused harm [19-21]. If successful, this could stimulate reporting by improving patient safety while generating new data on adverse drug events.

Without an understanding of adverse drug event reporting barriers, newly designed reporting software risks being ineffective [22]. To date, studies investigating underreporting have focused on provider knowledge and attitudes and advocated for interventions targeting provider behaviors [23,24]. They have framed underreporting as a failure of individuals without investigating work practice or system-level issues and have seen limited success [8,13]. Therefore, our objectives were to understand how adverse drug events are diagnosed and documented in clinical practice and examine barriers to reporting within existing systems to inform the design of software for adverse drug event reporting.

## Methods

### Design and Setting

We conducted a qualitative study using ethnographic workplace observations and focus groups between March 2014 and December 2016 in 1 rural ambulatory care center and in the emergency departments and wards of 2 urban tertiary and 1 urban community hospital in British Columbia, Canada [25,26]. The University of British Columbia Research Ethics Board approved the study protocol. We obtained verbal consent from participating health care providers and implied consent from focus group participants.

### Observational Fieldwork

Trained research assistants shadowed clinical pharmacists and physicians in emergency departments and on hospital wards during 4- to 8-hour data collection shifts. We scheduled shifts at varying times of the day and days of the week to account for variations in activity over time. We focused observations on pharmacists because identifying, documenting, and reporting adverse drug events are central to their role. We focused on emergency department settings because our prior work showed that patients with clinically significant events commonly present to emergency departments, where the diagnosis is often first suspected [2,15]. We recruited a convenience sample of participants through the contacts of clinicians on our team, email invitations, and word of mouth. We paid attention to the health care settings, presentations in which adverse drug events were suspected and managed, artifacts that mediated work (such as forms, computer applications, faxes, or phones), information flow, and interactions between clinicians. In this study, we use the terms “documentation” and “communication” of adverse drug events to refer to their recording for the purposes of providing clinical care, whereas we use the term “reporting” to refer to the activity of preparing and submitting a formal report to a pharmacosurveillance agency (eg, the MedEffect program in Canada or British Columbia’s Patient Safety Learning System).

### Focus Groups

We recruited a purposive sample of focus group participants from study hospitals, primary care offices, and community pharmacies in the Lower Mainland of British Columbia through team contacts, posters, and email invitations, targeting provider groups that encounter adverse drug events on a regular basis. We held 1-hour sessions at lunchtime rounds for participants practicing in-hospital and evening sessions for those practicing in other settings. We informed participants that our goal was to design a new electronic adverse drug event reporting system to reduce repeat events and improve reporting. The primary aim of the focus groups was to iteratively refine a set of data fields that would be relevant to clinical work and discuss the practicalities of diagnosing, documenting, and reporting adverse drug events. A practicing physician (CMH) and/or clinical pharmacist (KB) on our team led or co-led sessions while research assistants took field notes.

### Data Analysis

Two members of the project team (SSS and DP) independently coded observational field notes and notes from focus groups using NVivo 11 qualitative data analysis software (QSR International). After an initial review, we met regularly to discuss emerging findings and developed a formal coding structure (Multimedia Appendix 1). After coding all data, we performed an in-depth analysis by creating thematic summaries, workflow diagrams, and event summaries. We followed a qualitative descriptive approach to produce a description of the perceptions and experiences of our provider informants [27,28]. We iteratively presented interim findings from earlier focus groups and observations to later groups and to care providers to validate and contextualize our findings and refine data collection. We generated a set of generalizations based on the data collected, reflected on the practical application of our findings, and concluded observations and focus groups when they no longer yielded novel insights. Clinicians subsequently critiqued and validated our findings and provided examples of cases from their clinical work to illustrate the concepts we had identified.

## Results

### Data Collection

We completed 238 hours of observations with clinical pharmacists, including 197 hours in emergency departments and 14 hours on hospital wards, and 27 hours of observation with physicians in emergency departments. During our observations, providers investigated 65 cases of suspect adverse drug events. We held 7 focus groups with 85 care providers: 4 with hospital pharmacists, 1 with emergency department physicians, 1 with general practitioners, and 1 with hospitalists.

### Clinically Significant Versus Reportable Events

We observed care providers diagnosing a wide range of events (Textbox 1). Many were not categorized as adverse drug reactions but were categorized as dosing problems, noncompliance, treatment failures, ineffective drugs, drug interactions, untreated indications, and drug use without an indication. None of the observed events was due to errors in drug ordering, transcribing, dispensing, or administration. Events could often be categorized in various ways. For example, a seizure related to the coprescription of 2 drugs could have been categorized as an adverse drug reaction, a drug-drug interaction, or a prescribing error (Textbox 1, example 3).

Providers generally made their own judgments about what events should be documented, with some rejecting the use of the term adverse drug event for events related to nonadherence or suboptimal dosing (Textbox 1, examples 2 and 6).

**Examples of adverse drug event categorizations deemed clinically significant.**

Low-dose adverse drug events:

- Example 1: When a patient presented with swelling in her legs, the pharmacist observed the patient had been newly prescribed a diuretic. The pharmacist noted that the choice of diuretic and the dose were reasonable but the dose was likely ineffective. The patient had been prescribed too low of a dose and, as a result, developed symptoms that brought her to the emergency department **.**
- Example 2: A patient diagnosed with high blood pressure and atrial fibrillation had been taking the oral anticoagulant warfarin at prescribed doses but presented to the emergency department with a low international normalized ratio (INR; a measure of the effect of warfarin) and an ischemic stroke. This was thought to be due to the low dose of warfarin the patient had been taking, leading to a subtherapeutic INR.

Drug interactions:

- Example 3: A patient presented to the emergency department with a seizure after having taken buproprion, citalopram, and clonazepam. The pharmacist noted that buproprion alone could have caused seizures at high doses; however, the patient was taking a low dose. The pharmacist then discovered the reported seizure risk was highest when buproprion was taken together with other antidepressants such as citalopram. After a negative workup for other causes, the pharmacist and physician concluded that an adverse drug event from the coingestion of multiple medications was possible.
- Example 4: A patient with a history of atrial fibrillation, stroke, and seizures presented to the emergency department with new neurological deficits. Imaging revealed a new stroke. During the patient’s hospitalization, the pharmacist discovered the patient had recently been started on phenytoin to treat his seizures. This drug interacted with the patient’s anticoagulant, dabigatran, which had been prescribed to prevent further strokes, and reduced dabigatran’s anticoagulant effect. This drug interaction was likely the cause of the patient’s recurrent stroke.

Nonadherence:

- Example 5: A patient presented to the emergency department with a seizure after having missed some doses of the anticonvulsant carbamazepine. The patient was unsure of how many doses they had taken during the week and was evasive in responding to the pharmacist’s questions. Alternative diagnoses were ruled out.
- Example 6: A patient with a history of atrial fibrillation who had been prescribed dabigatran for stroke prevention presented to the emergency with left-sided face, arm, and leg paralysis and was diagnosed as having suffered a large ischemic stroke. The patient reported that he had missed 1 dose of dabigatran the night before.

Regardless of the variability in terminology used, all types of events were important to patients, their caregivers, and clinicians, as adverse drug event symptoms were often uncomfortable, could be associated with permanent disability, required changes to the patient’s management, and often resulted in hospital admission or additional health care visits. Despite the clinical relevance of a broad range of adverse drug events, pharmacists were often uncertain about which ones to report to external agencies.

Finally, some care providers were concerned about using the term adverse drug event for events that were an expected part of clinical care or had previously been described in the literature. Others used the term adverse drug event to refer to preventable events, rejecting the term when a patient experienced a nonpreventable predictable side effect. Severity also affected how providers characterized events. In one case, the pharmacist was reluctant to classify hyponatremia due to a thiazide diuretic as an adverse drug event even though the medication had to be withdrawn to prevent deterioration (Table 1, example 7).

### Challenges in Diagnosing Adverse Drug Events

We identified several sources of complexity when care providers diagnosed adverse drug events (Table 1). We witnessed care providers making difficult, context-specific decisions while managing high-acuity patients in high volumes with frequent patient turnover, limited time, and many interruptions.

The medication and medical histories were often limited or uncertain at the time care providers made prescribing recommendations or decisions (Table 1, examples 1 and 2). As a result, care providers often managed patients based on a working rather than definitive diagnosis (Table 1, example 5).

Care providers diagnosed adverse drug events over time and often across different settings. While one care provider may have suspected the adverse drug event and held the medication, a different care provider may have confirmed the event (Table 1, examples 3 and 4). In 45 of 65 suspect adverse drug events (69%), the diagnosis could not be confirmed before the end of the initial provider’s shift, and the patient required follow-up to confirm the event and guide further management (Table 1, examples 3 to 5). Often, more than one type of complexity compounded the difficulty in confirming adverse drug events.

Providers took ad hoc and informal approaches to coordinate and ensure follow-up of suspect events, making phone calls or sending faxes to outpatient providers or instructing patients and caregivers to follow up with their physician or outpatient pharmacist. Clinicians identified that inadequate monitoring and follow-up and informational discontinuity of care posed a risk to patients. These problems could arise at handovers at the end of their shift, between provider groups, or across health settings if the patient was discharged. Establishing continuity of care was identified as challenging. As one pharmacist expressed, “This is one of the challenges with adverse drug events in the emergency department. Once the patients leave, it’s not entirely clear what happens in their care.”

### Documentation

We observed providers document 41 adverse drug events in clinical charts. They documented to record their clinical assessments, justify a therapeutic action, or ensure informational continuity of care. Events were documented in site-specific electronic or paper-based medical records (Table 2). In general, pharmacists faxed care providers in the community when they felt the patient was at risk of reexposure or when it was important to notify the patient’s physician. British Columbia’s provincial electronic medication dispensing database, PharmaNet, allows for free-text information on adverse reactions to be recorded within a patient’s profile. However, few providers have access to enter this information, and we observed only 3 instances in which a pharmacist documented in PharmaNet. Care providers criticized the inflexible design of reporting options as being restrictive and incompatible with the complex nature of many adverse drug events, noting limited dropdown menus, dosing options, and character counts. One pharmacist noted, “sometimes the complex real story just doesn’t fit; there’s nowhere to specify the ifs, ands, or buts.” In addition, reporting systems did not allow for reports to be changed, updated, or removed when new information became available over time or in different health care settings (Table 1, examples 3 to 5), making pharmacists reluctant to use electronic reporting forms even when they were certain about the diagnosis. Providers found documenting complex adverse drug events to be less problematic when writing in clinical notes, where they could structure their own notes and make reference to contingencies, follow-up requirements, and uncertainty.

**Figure 1:**
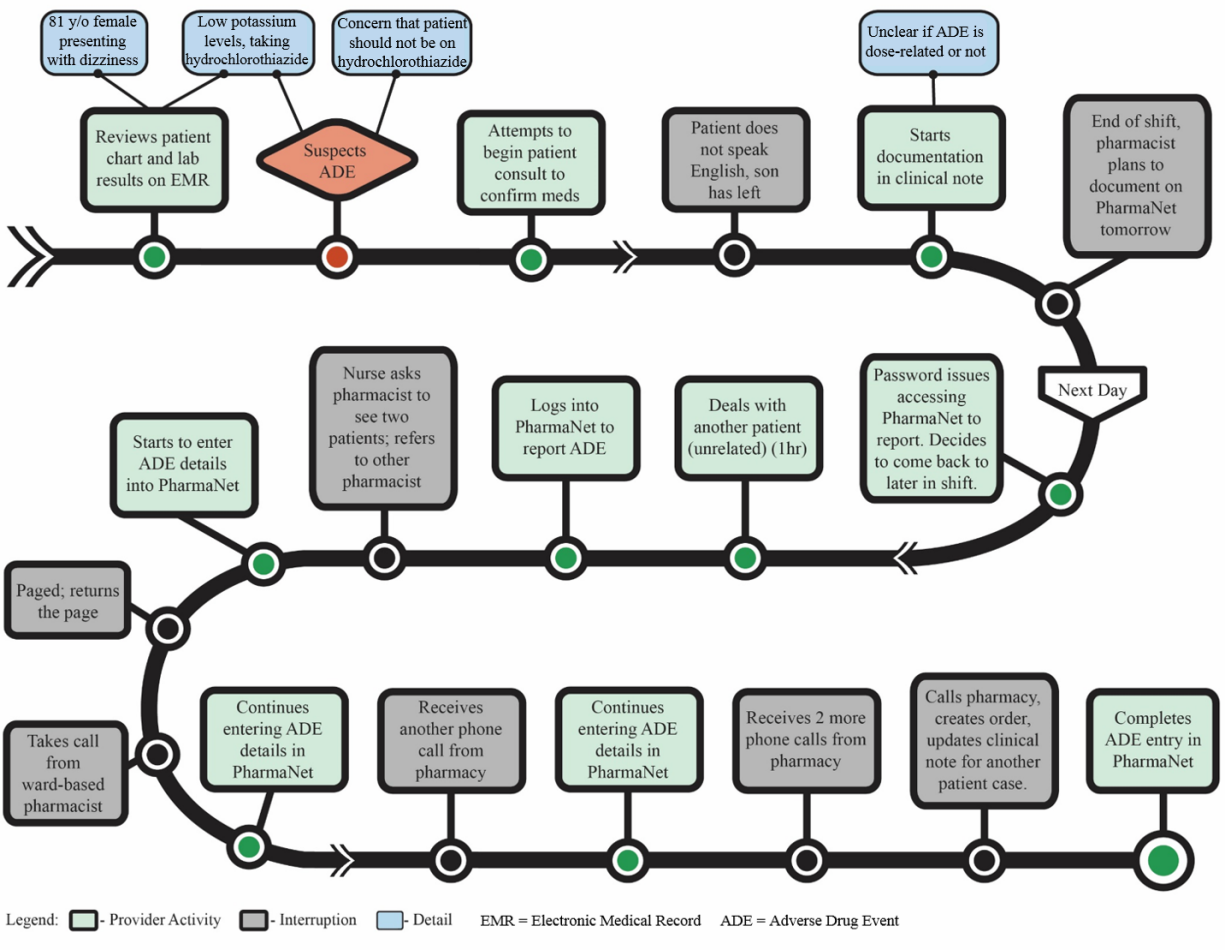
Clinical pharmacist's workflow documenting an adverse drug event.

Time pressures influenced the extent to which care providers documented as they commonly managed multiple patients simultaneously. They were regularly interrupted and were often busy or off shift when information required to confirm an adverse drug event became available (Figure 1). Emergency physicians reported suffering from near-constant interruptions. One emergency physician commented: “I want to give [the patient] to someone who has more time than me,” and “I’m still waiting on phone calls so I’ll be interrupted again—it’s killing me.” Adding to this, the documentation process was itself time-consuming. In order to diagnose and document adverse drug events, care providers needed to search multiple sources for relevant information (eg, medication dispensing information, laboratory tests). The consequence of time pressures was that demands related to providing immediate patient care took precedence over documentation, which in busy times was often delayed, incomplete, or not completed at all.

**Table 1:** Complexities in diagnosing or refuting adverse drug event diagnoses.

Complexity description		Examples (from observation field notes)
**Medication history uncertain**		
	Obtaining an accurate medication history and establishing the timeline between medication exposure (or lack thereof) and symptom onset is challenging and makes it difficult to recommend changes to the medication regimen.	Example 1: A patient presented to the emergency department with rectal bleeding. The pharmacist discovered that the patient’s INR^a^ was too high, indicating an adverse drug reaction or a high-dose adverse drug event. The patient showed the pharmacist 2 bottles of warfarin (one in 3-mg dose from August 2014 and another in 4-mg dose from January 2013) and said he could not remember which dose he had been taking and that he might have been alternating between 3 mg and 4 mg doses every other day. The patient’s other drugs were blister packed, and upon inspection the pharmacist found that the patient was also nonadherent with the other drugs. This made it difficult to ascertain the dose of warfarin that led to the patient’s high INR, making dosing adjustment challenging. In addition, the patient’s INR could have been elevated for some time and just hadn’t been measured. This made recommending a new warfarin dose challenging.
**Lack of adequate definitive information**		
	Time constraints, incomplete documentation within a medical record, and inability to recall information may make an adverse drug event assessment impossible	Example 2: A patient with chest pain was seen by a pharmacist in the emergency department. The patient was confused and could not describe their medications or the timeline of symptom onset. The confusion had not previously been documented in the hospital record. The patient was from a long-term care facility where care providers administer medications, but the medication administration record was not available so the pharmacist could not verify the medications. Assumptions about this patient’s medication use had to be made while managing him according to a working diagnosis. While an adverse drug event was possible, the pharmacist could not obtain sufficient information to refute or confirm an adverse drug event diagnosis.
**Diagnostic evolution over time**		
	The signs and symptoms of adverse drug events develop and are diagnosed over time, often involving multiple care providers and care settings. Definitive diagnostic test results may not be available, and care providers may have to manage patients according to a working diagnosis.	Example 3: A patient recently finished a course of antibiotics to treat pneumonia, but the cough persisted. The pharmacist suspected that the patient’s relatively new prescription of ramipril may have been contributing to the cough but was uncertain given that the cough developed before the patient began taking ramipril. Causality was uncertain, but after consultation with the physician, ramipril was changed to an alternative agent. The pharmacist faxed the patient’s general practitioner to request follow-up for this patient to determine whether the cough persisted after the change in medication. Only the general practitioner would be able to confirm the adverse drug event diagnosis if the cough persisted despite resolution of the infection.
		Example 4: A patient presented to the emergency department with diarrhea, having recently been on amoxicillin–clavulanic acid to treat a dental infection. The patient was well enough to go home, but the diagnostic test to confirm *Clostridium difficile* colitis was still pending. The patient’s family physician would make the definitive diagnosis.
		Example 5: A patient presented to the emergency department vomiting blood. The patient had been on naproxen, a drug that can cause gastrointestinal inflammation and ulcers. On endoscopy, the patient was diagnosed with a gastric ulcer that was attributed to naproxen and managed accordingly. However, biopsy results that became available several weeks later revealed a gastric adenocarcinoma, thus refuting the previous diagnosis of an adverse drug reaction.
**Causality assessment**		
	Complex presentations make assessment of causality uncertain. It can be difficult to distinguish whether symptoms are due to an adverse drug event or an exacerbation of a preexisting medical condition.	Example 6: A patient presented to the emergency department with suicidal ideation. About 1 week prior, the patient decided to stop the antidepressant and antipsychotic medications trazodone, methotrimeprazine, and quetiapine hoping to increase their energy level. The patient expressed being under high stress related to a cockroach infestation in the home and concerns over their father’s health. It was unclear whether stopping the medications or the patient’s extenuating circumstances caused the patient’s deterioration.
**Expectedness of event impacts propensity to document and report**		
	Providers may not consider documenting and reporting adverse drug events for mild, frequently encountered, or expected adverse effects.	Example 7: A patient was hyponatremic due to the prescribed diuretic indapamide. Prior to the patient's discharge, the clinical pharmacist advised the patient and family member about discontinuing the drug and suggested that the patient follow up with his general practitioner. After the patient consult, the pharmacist noted that other providers may interpret the term adverse drug event differently and may assume that a documented adverse drug event means that the drug is contraindicated. She noted that, given the particulars of this case, she thought that the patient’s low sodium was something to be expected and was not critical to communicate directly with the general practitioner. She documented the event in her clinical note.
	Providers may suspect an adverse drug event but not consider it worthy of documentation or reporting if the presenting signs and symptoms have not previously been described as being related to medication use.	Example 8: A patient presented to the emergency department with a subarachnoid hemorrhage, a life-threatening neurosurgical emergency. The patient did not report a preceding head injury or history of migraines but had taken ergotamine. The pharmacist speculated that the subarachnoid bleed may be related to the patient’s use of ergotamine. Ergotamine causes vasoconstriction and raises blood pressure, which the pharmacist hypothesized could have contributed to the subarachnoid hemorrhage. The pharmacist could not find conclusive evidence linking ergotamine to subarachnoid hemorrhage, so she decided not to document or report the event as a suspect adverse drug event.

^a^INR: international normalized ratio; a measure of the effect of the oral anticoagulant warfarin.

**Table 2:** Modes of adverse drug event documentation and communication observed.

Type of documentation and communication	Total instances^a^
Paper chart	35
Electronic chart	14
Fax to general practitioner	6
PharmaNet	3
Community pharmacy system^b^	2
MedEffect Canada	0
Not documented	24

^a^More than one instance may have occurred per event; therefore, total instances of documentation exceeds total number of observed events.

^b^Provider called community pharmacy to have a note added to the patient’s profile within the pharmacy system.

**Sample comments from providers related to documentation burden.**

- “A huge barrier [for electronic reporting] is the time it takes for the process of getting from opening my [office] door to getting this screen open in front of me.” [hospitalist]
- “Motivators to report are the simplicity of [a new reporting] tool, the fit into clinical workflow or processes, and the time taken to complete a report.” [emergency department physician]
- “I don’t have time to take ownership [of routine reporting], but I would happily collaborate with the pharmacists and do my part in it.” [hospitalist]
- “The more fields, the less likely I am to enter it.” [clinical pharmacist]
- “[Reporting] is duplicative and potentially quite onerous, especially for older patients who are on a number of medications.” [hospitalist]
- “There won’t be any buy-in if there’s too much stuff to fill out.” [general practitioner]

### Reporting

Our team never observed a pharmacist or physician report to an external agency such as Canada’s MedEffect program. Care providers viewed reporting to regulatory agencies as a burden and told us that convenience, speed, and simplicity should be central design considerations for new reporting tools (Textbox 2). In order to report adverse drug events, they noted that they would have needed to search for paper or online forms external to their facility’s electronic medical record which demanded time they did not have. Furthermore, these forms typically contained around 35 data fields and collected information that they had already documented in the patient’s record, representing duplication of work.

## Discussion

### Principal Findings

Our objective was to understand how adverse drug events were diagnosed and documented in hospitals and examine barriers to reporting. We found a high degree of complexity and uncertainty in diagnosing adverse drug events in clinical practice. Despite these challenges, care providers regularly documented events as part of their work to provide informational continuity of care. However, they never used existing electronic reporting systems, which would have required data entry at one point in time by a single individual and did not reflect the complexity of the clinical diagnosis or the providers’ workflow. Clinicians faced time constraints and perceived existing reporting systems as an additional documentation burden, requiring time to access and representing a duplication of tasks without providing additional benefit to the patient.

Our results confirm that clinicians are interested in documenting and reporting adverse drug events and would welcome reporting mechanisms that meet clinical needs while allowing them to observe the direct impact of reporting on clinical care. For example, clinicians on our study spent an inordinate amount of time attempting to contact other care providers (eg, by phoning or faxing) to ensure that adverse drug events were communicated to other care providers. Electronic reporting systems could facilitate communication by automating the electronic communication of standardized adverse drug event reports between clinicians or creating patient-level alerts to ensure that other care providers do not inadvertently reexpose patients to culprit drugs. This form of feedback was perceived as highly relevant and would motivate the use of a reporting system.

Previous studies drew on a theoretical model that outlined a set of provider attitudes, including complacency, indifference, and ignorance, to which underreporting was ascribed [29-31]. Studies that used this model have been questionnaire- or interview-based and implied that providers neglect their responsibility to research, safety, and regulatory agencies and the public by not reporting [23,24,32-34]. As a result, attempts to improve reporting have focused on using incentives, education, legislation, or guidelines to correct provider behaviors [35]. While some of these initiatives have led to short-term local improvements, adverse drug event underreporting remains problematic [8,13]. Initiatives aimed at improving reporting would likely benefit from a similar paradigm shift as the patient safety movement, which has moved away from a culture of individual blame toward system-level analysis to examine and improve organizational structures and technology design [36,37].

In contrast to prior studies, our approach offers a qualitative analysis of the real-world management and documentation of adverse drug events. Our findings suggest that failure to document and report is a system-level problem that might more successfully be solved by redesigning reporting systems to address the practical concerns of care providers, assist them in their clinical work, and reflect the complex nature of adverse drug events. We suggest an approach that builds on new capacities offered by electronic medical records, which are now widely used. In contrast to existing reporting systems that are oriented to data collection for research and regulatory purposes, systems might be repurposed to facilitate documentation and information flow between care providers and across health sectors (eg, between ambulatory care settings, hospitals, and community pharmacies), addressing major concerns for care providers [21]. Such systems may reduce the risk of reexposures to harmful medications while generating high-quality adverse drug event data for surveillance and research.

Our team developed 5 core recommendations in order to mitigate system-level issues in adverse drug event reporting. First, in order to ensure uptake and utility for clinical care, reporting systems must act as a mechanism to document work and share information between care providers. This approach minimizes duplication of work. By linking patient-level adverse drug event reporting to clinical documentation and enabling communication to prevent harmful reexposures, new approaches may motivate care providers to report events. Second, by integrating adverse drug event reporting into existing electronic interfaces, the time and barriers (eg, multiple passwords) required to access reporting forms can be minimized, and fields can be autopopulated with readily available information to minimize data entry. Third, systems that clinical care providers are expected to report in should only include data fields relevant to clinical practice. Fourth, systems should enable standardized and categorized data entry to speed up reporting and enable standardized data to be generated while allowing free-text entry in other locations so care providers can document nuanced information for complex events. Fifth, adverse drug event reports should be living documents that enable multiple providers to edit, update, and remove data as information becomes available or a patient’s condition changes.

### Limitations

Our findings reflect the activities and opinions of pharmacists and physicians working in the settings where we were able to conduct the study. While many care providers had worked outside of these institutions and in other provinces prior to our project, the generalizability of our findings to other clinical areas and jurisdictions may be limited, as environmental conditions, work organization, information infrastructures, culture, and job tasks vary across facilities and jurisdictions. Finally, we sought to explore and describe barriers to underreporting but found reporting to external agencies to be so rare that we were unable to observe any reports being created for external agencies. Our focus was on adverse drug events and not on patient safety incidents or errors, as these types of events cause a minority of adverse drug events in our clinical setting.

### Conclusion

While providers routinely document adverse drug events in clinical records to inform patient care, barriers exist to report to external agencies. We recommend that future reporting systems are designed to enable providers in documenting and communicating adverse drug events as ambiguous, unfolding, and uncertain events and help clinicians meet patient safety goals. Integrating such reporting systems into electronic medical records could alleviate time pressures for clinicians and may produce more robust and complete adverse drug event data as a by-product of safer clinical care.

## Appendix

Multimedia Appendix 1 Coding structure.

## Abbreviations

INR: international normalized ratio
