# Supplementary material for: Why Clinicians Don’t Report Adverse Drug Events: Qualitative Study
Source: JMIR Public Health Surveill. 2018 Feb 27;4(1):e21. doi: 10.2196/publichealth.9282 (PMC5849794; doi:10.2196/publichealth.9282)
Supplement: Multimedia Appendix 1 [file publichealth_v4i1e21_app1.pdf]

Coding Scheme

**Uncertainty**	Provider is/appears uncertain of diagnosis or action (e.g., is this an ADE? What type? Is it resolved?)
⎣ Diagnostic	Anything related to "Is this an ADE?"
⎣ Type	Provider is/appears uncertain regarding determination of the type of ADE. (e.g., multiple types, ambiguity)
⎣ Causality	Provider is/appears uncertain about the causality of the ADE
⎣ Resolution	Provider is/appears uncertain because event is unresolved in the ED.
**Shift to Certainty**	Provider has resolved their uncertainty and has assumed a definite position on the ADE.
**Provider Updates/Advises Another Provider**	Provider advises another provider of their action or thoughts regarding a patient (e.g., talks to ED physician, faxes a GP, phone community pharmacist)
**Professional Boundaries**	Provider indicates/describes his/her or other's role in the ED/hospital/process of patient care (e.g. professional identity). Also include personal reflections.
**Information Source**	
⎣ *Internal*	*Sources internal to the hospital*
⎣ Patient / patient family consult	Provider conducts a consultation with the patient/family to gain information on the patient. Include artifacts that the patient or family has brought with them.
⎣ PharmaNet	Provider accesses/refers to PharmaNet (electronically or on paper)
⎣ PCIS	Provider accesses/refers to PCIS on computer
⎣ Paper chart	Provider refers to the patient’s paper chart
⎣ Medrec form	Provider refers to the patient’s medrec sheet
⎣ Lab data	Provider accesses/refers to the patient’s lab data (electronically or on paper)
⎣ Patient transcripts	The provider accesses or refers to the patient’s current or past patient transcripts (e.g. discharge summaries, in-hospital summaries, etc.)(electronically or on paper)
⎣ Provider-provider	Provider gains information from another care provider within the hospital (*code to provider type*)
⎣ ED Physician	
⎣ ED Pharmacist	
⎣ Nurses	
⎣ Specialist	
⎣ Other	
⎣ Other	Provider accesses any other information source within the hospital
⎣ *External*	*Sources external to the hospital*

⎣ Source	Provider refers to non-human information source (e.g., websites)
⎣ CareConnect	Provider accesses patient's CareConnect profile.
⎣ MAR	Provider refers to a patient's MAR
⎣ Provider-Provider	Provider gains information from another care provider who is external to the hospital (*code to provider type*)
⎣ GP	
⎣ Community pharmacist	
⎣ Other	
Mode	*Mode of access for information source*
⎣ Computer-based	Source is accessed on a computer
⎣ Paper-based	Source is paper-based
⎣ Phone (internet)	Source is accessed using the internet capabilities of a phone
⎣ Phone (call)	Source is accessed through a phone call
⎣ Face-to-face	Information gathered through a face-to-face interaction.
**Workflow Process**	
⎣ Challenges	Challenges relating to workflow/care processes relevant to ADEs.
⎣ Artifacts	Challenges related to non-human artifacts (e.g., computers, software, pill bottles)
⎣ People	Challenges related to people (e.g., provider fatigue, patient language, patient cooperation, patient memory, collaborative break, provider disagreement, reluctance to interrupt)
⎣ Information	Challenges related to information (e.g., unable to access info, had to find a workaround, data quality, incomplete records)
⎣ Workspace	Challenges related to the physical space where provider is working (e.g., unfiled patient documentation, access to computers)
⎣ Time pressure	Challenges related to time (e.g., provider too busy)
⎣ Financial/administrative pressures	Financial or administrative pressures affect the provider or the provider talks about these pressures (e.g., pharmacist paid to dispense but not consult)
⎣ ADE documentation practices	Provider talks about or performs current ADE documentation practices (e.g., records an allergy in PCIS, faxes a GP, makes a note in PharmaNet)
⎣ Note-taking	Provider engages in informal note-taking processes (e.g., on Clinical Notes sheet)
⎣ Information trajectory	Disposition of information trajectory (e.g., occasions when documentation does not result in effective communication)
**General Issues**	
⎣ Privacy issues	Issues relating to provider or patient privacy
⎣ PharmaNet limitations	Issues relating to limitations of PharmaNet (e.g., certain meds do not appear, not updated)
⎣ Legal Implications	Issues relating to legal implications for providers

**Factors Affecting Decision to Report**	
⎿ Problematic reporting	Provider is/appears/talks about uncertainty about reporting an event because of its implications (e.g., an inappropriate dosage today may be appropriate in future, provider doesn't want to "blacklist" a medication for the patient, non-adherence may be offensive to patient, event too complex to describe adequately in short report, etc.)
⎿ Beliefs and attitudes	Provider expresses/demonstrates beliefs/attitudes about reporting ADEs (e.g., only report serious events)
**ADE Type**	*Record the type of ADE when an ADE is described or occurs, or relevant information on a specific type of an ADE is given. Include historical ADEs that are being described to the observer.*
⎿ ADR	An event that may be the result of an ADR.
⎿ Allergy	An event that may be the result of an allergy
⎿ Dosage Issues	An event that may be the result of a subtherapeutic or supratherapeutic dose.
⎿ Drug-drug interaction	An event that may be the result of multiple drugs interacting.
⎿ Duplicate therapies	An event that may be the result of a patient being prescribed multiple drugs for the same indication.
⎿ Illicit drugs	An event that involves illicit drugs.
⎿ Incorrect/wrong drug	An event that may be the result of a patient being prescribed a drug of questionable efficacy for their indication, perhaps as therapeutic trial or based on a working diagnosis. Also for cases where an unusual, older, or a drug of lower efficacy than others is prescribed.
⎿ Patient non-adherence	An event that may be the result of a patient's non-adherence to a medication (include voluntary and involuntary non-adherence)
⎿ Provider error	An event may be the result of a provider's error in prescribing, dosage, administration, monitoring, etc.
⎿ Side effect	An event that may be a side effect of a medication.
⎿ Treatment Failure	An event that may be the result of a treatment failure.
⎿ Multiple type	An event that can be characterized as more than one of the above types *(code as each type and as "Multiple type")*
**ADE Definition Issues**	Coder, provider, or observer note a definitional issue at play (e.g., provider unsure of whether to characterize medication error / side effect as ADE)
**Personal Comment**	*Any time a provider, patient, observer offers an opinion.*
⎿ Suggestion	Provider or observer suggest an improvement/adjustment (e.g., workflow changes, data fields)
⎿ Anecdote	Provider or patient offers an anecdote.
⎿ Questions	Provider or observer poses a question
⎿ Gold Quote	Remarkable, insightful, astute quotes! Meaningful comment from patient/provider.

⎣ Subject	Personal comment is offered by observed provider (*code as opinion type and 'subject'*)
⎣ Observer	Personal comment is offered by observer (*code as opinion type and 'observer'*)
**History (Past Event)**	Provider discusses an ADE that has previously occurred (*code as ADE type and 'history'*)
